# Revolution of hands-on model for interventional EUS: How to make a training model

**DOI:** 10.1097/eus.0000000000000046

**Published:** 2024-01-23

**Authors:** Tanyaporn Chantarojanasiri, Aroon Siripun, Ratchamon Pinyotheppratarn, Pradermchai Kongkam, Nonthalee Pausawasdi, Thawee Ratanachu-ek

**Affiliations:** 1 The Thai Association for Gastrointestinal Endoscopy (TAGE), Bangkok, Thailand; 2 Division of Gastroenterology, Department of Internal Medicine, Rajavithi hospital, College of Medicine, Rangsit University, Bangkok, Thailand; 3 Department of Gastroenterology, Bangkok Hospital, Bangkok, Thailand; 4 Department of Surgery, Rajavithi hospital, Bangkok, Thailand; 5 Excellence Center for Gastrointestinal Endoscopy and Division of Gastroenterology, Department of Medicine, Faculty of Medicine, Chulalongkorn University and King Chulalongkorn Memorial Hospital, Thai Red Cross Society, Bangkok, Thailand; 6 Division of Gastroenterology, Department of Medicine, Faculty of Medicine Siriraj Hospital, Mahidol University, Bangkok, Thailand.

**Keywords:** EUS intervention, Model, Training

## Abstract

EUS interventions have an increasing role in the treatment for hepatobiliary-pancreatic diseases. However, the procedure itself is not frequently performed, needs expertise, and carries a high risk of complications. With these limitations, the hands-on practice model is very important for the endoscopist in training for EUS intervention. There have been various hands-on models for EUS interventions, ranging from *in vivo* living pig model to all-synthetic model. Although a living model provides realistic sensation, the preparation is complex and increases concerns for zoonotic issues. All-synthetic models are easier to prepare and store but not realistic and still need the room for improvement. Hybrid *ex vivo* model is more widely available and provides various training procedures but still needs special preparation for the porcine tissue.

## INTRODUCTION

EUS has an increasing role for the diagnosis and treatment of gastrointestinal diseases. Since its first development in 1980s, the role of EUS has been changed from purely diagnostic indications to therapeutic interventions for organs that are located adjacent to the gastrointestinal tract. In some conditions such as well-formed pancreatic fluid collection (pseudocyst) or failed transpapillary approach in biliary obstruction, interventional EUS is now replacing surgical interventions or percutaneous approach as the first-line treatment.^[1]^ However, EUS-guided therapy is still technically demanding and carries a high complication rate. EUS-guided intervention is not frequently performed^[2]^ even in a tertiary care center. To become more familiar with the procedure, various kinds of models have been developed to improve the endoscopic skill as well as the bridge to development of new procedure. Live swine model has been used for the EUS training because most of internal anatsomy is similar to human.^[3,4]^ However, it is expensive and infeasible for repeated procedure and needs a dedicated endoscope due to the concern of zoonotic issues. Moreover, multistep procedures on the live pig, such as the creation of biliary obstruction or target for biopsy, are required to prepare the condition suitable for EUS-guided intervention.

This review summarizes various kinds of models used for EUS training and research, ranging from *ex vivo* model to the all-synthetic model. The aim of this review is to provide more knowledge of how to set up the interventional EUS model for educational purposes.

## MODEL FOR EUS FINE-NEEDLE ASPIRATION/FINE-NEEDLE BIOPSY

EUS fine-needle aspiration (FNA)/fine-needle biopsy is the procedure that aims to achieve tissue diagnosis in the organ adjacent to the EUS probe. The technique consists of stable scope position, lesion identification, needle insertion, and puncture technique. The training model for EUS-FNA provides practice in the puncture techniques, with limited practice on keeping stable scope position or lesion identification.

### Tissue model

Tissue models in live pig have been very useful for EUS-FNA training as it provides similar echogenicity and texture as in human. To make a target for biopsy, autologous blood mixed with carbon particles was injected into the mediastinal lymph nodes and celiac lymph nodes under EUS guidance. EUS-FNA training was performed 2 weeks after the injection.^[5]^

Hoshi et al.^[6]^ reported the *ex vivo* tissue model using pig stomach combined with chicken breast for practicing FNA. The tissue model provided a good texture during EUS-FNA and had good echogenicity. However, using porcine tissue necessitates immediate preparation before the procedure and requires good storage.

### Nontissue model

**Figure 1:**
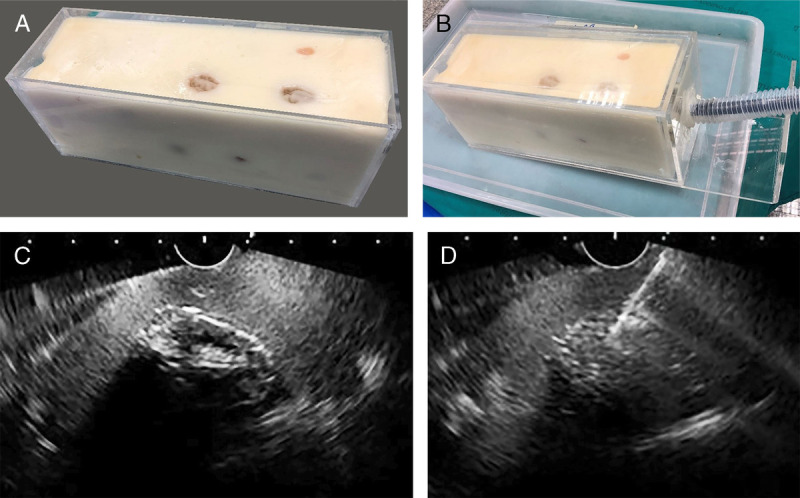
EUS-FNA model using gelatin-cornstarch mixture (A) placed in acrylic box (B). Peeled grape could be placed to simulate retroperitoneal lymph node (C) or, alternatively, an egg yolk dessert to create hyperechoic lesion as a target for EUS-FNA (D). FNA: Fine-needle aspiration.

The nontissue models for ultrasound-guided FNA have been developed for years. Reports of EUS-FNA models using concentrated gelatin with or without mixture with cornstarch has been published.^[7,8]^ Cornstarch mixture will help increase the echogenicity of the material to mimic real tissue.^[7]^ The same principle can also be applied in the EUS-FNA model from ours as shown in Figure 1. The gelatin mixed with cornstarch was used to represent retroperitoneal tissue, and peeled grapes were embedded to make a target for biopsy [Figure 1A]. The model was placed in an acrylic box with corrugated tube attached for scope insertion [Figure 1B]. Various kinds of target lesion could be decided, depending on the material inserted. For example, grapes and tomato are used to create hypoechoic lesion [Figure 1C], whereas Thai sweet made from egg yolk could provide hyperechoic lesion due to high fat content [Figure 1D]. The model could be prepared as follows: mix 120 g of cornstarch and 140 g of gelatin with 700 mL of normal saline in a heat-resistant container. Place the container in the steaming pot for 10 minutes, and leave the steaming pot for cooldown for 20 minutes. Stir the mixture well and pour into the mold. Keep the mold refrigerated at 4°C for 45 minutes.

This model has a good echogenic view and is inexpensive but still has some limitations because it needs to be refrigerated and easily spoiled.

## MODEL FOR EUS PSEUDOCYST DRAINAGE

EUS-guided pseudocyst drainage can generally be performed by using either plastic stents or metal stents. The drainage procedure with plastic stent consists of multiple steps starting from identification of puncture site under EUS view, needle puncture, fluid aspiration, guidewire coiling inside the lesion, tract dilation, and stent placement. In case of using lumen-apposing metallic stents (LAMSs), the steps of guidewire coiling and tract dilation could be omitted in cautery-tipped LAMSs but still need in non–cautery-tipped LAMSs. Training models for EUS-guided pseudocyst drainage can provide the step practice from cyst identification to stent placement in both tissue and nontissue models.

### Tissue model

In 2006, Schofl et al.^[9]^ reported using a modified version of the Erlangen Active Simulator for Interventional Endoscopy model, which was initially developed for endoscopic retrograde cholangiopancreatography for practicing EUS-guided pseudocyst drainage. This model consists of explant pig esophagus, stomach, duodenum, and biliary system. The gallbladder was filled with water mixed with blue dye by transpapillary catheter and attached to the stomach by suturing. By this method, only endoscopic-guided transmural puncture, guidewire coiling, and plastic stent insertion were applicable. Later, a model using pig colon filled with gelatin and, sometimes, tapioca pudding to simulate necrotic tissue attached to the pig stomach was reported to provide similar puncture sensation and could withstand multiple punctures, tract dilation, and metallic stent insertion.^[10]^

### Nontissue model

**Figure 2:**
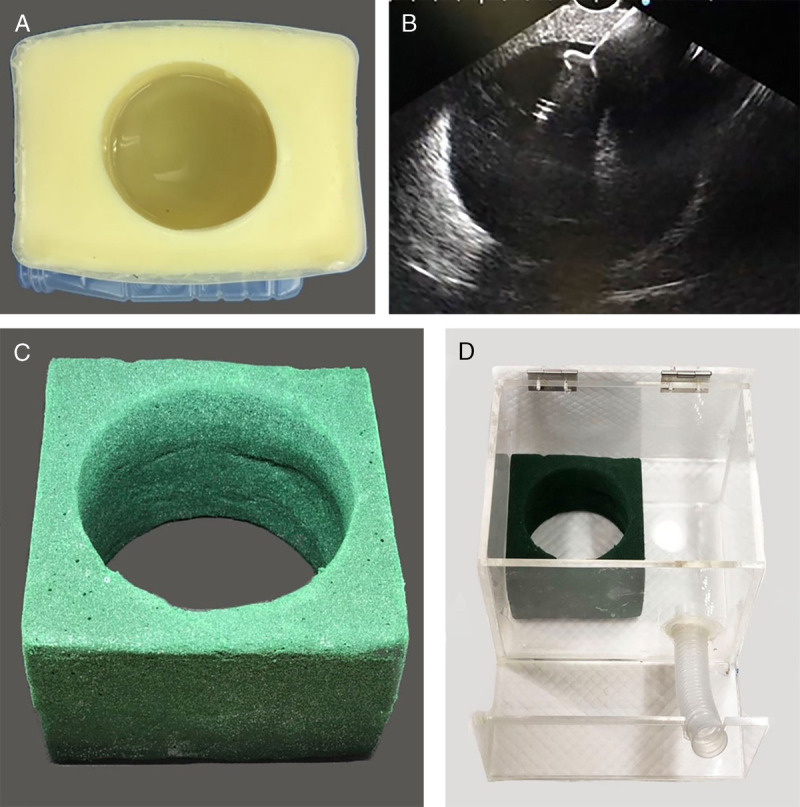
All-artificial model for EUS pseudocyst drainage. The early version was mode of gelatin-cornstarch mixture (A), which provided good echo image during endoscopic procedure (B). This model was less durable than the floral foam (B), which was placed in acrylic box filled with water (C).

All nontissue models for EUS pseudocyst drainage using gelatin and cornstarch were first presented in 2017 by Pinyoteppratarn et al.^[11]^ [Figure 2A], which are simple and can be adapted into other procedures such as choledochoduodenostomy (CDS) and hepaticogastrostomy (HGS). The model needs to be submersed in water and filled in transparent plastic box prior to the usage. The water filled in the center cavity to represent the pseudocyst and the instrument is directly visualized through the transparent plastic cover. The echogenicity and the visibility of the instrument are shown in Figure 2B. This model is easy to prepare and can be cauterized. However, the major drawback for this model is that it is not durable and needs appropriate storage. Later, the same group developed another model using a floral foam [Figure 2C] placed in acrylic box [Figure 2D], which was more durable and needed less storage condition. The floral foam needs to be submersed in water prior to the preparation to get rid of air trapping inside the foam and enable cauterization. This floral foam is later shaped as a cube with a central cylindrical hole, which, when filled with water, will provide the round echo that represents the pseudocyst. This provides the direct visualization of the instrument during the procedure but lacks realistic sensation for wire coiling.

## MODEL FOR EUS BILIARY DRAINAGE

EUS biliary drainage (EUS-BD) has been accepted as an alternative treatment for biliary obstruction after a failed transpapillary approach.^[1]^ Compared with EUS-guided pseudocyst drainage, EUS-BD procedure has more complexity and carries a higher rate of complications. To overcome the learning curve, up to 32 cases are needed, and more than 100 cases are needed to become mastery.^[12]^ As a result, the EUS model is very useful to improve the maneuverability and practicing the steps for the trainee.

The common EUS-BD procedures are EUS-HGS and EUS-CDS. The selection of these procedure depends on the anatomical factors, level of obstruction, and technical expertise.^[13]^ The procedure could be performed either as transmural stent placement, rendezvous procedure, or antegrade stenting.^[14]^ Steps in EUS-BD started with puncture site selection, guidewire insertion into selected part, tract dilation, and stent placement.^[13,15]^ In case of rendezvous procedure, the guidewire is inserted through the ampulla and retrieved by duodenoscope.^[15]^ Most training models focus on transmural stenting, with emphasis on the puncture, wiring, dilation, and stent placement.

### In vivo tissue model

Because the anatomy of pancreatobiliary system in swine model is similar to that in human, EUS training using swine model has been adopted for EUS training.^[3]^ However, for EUS-BD procedure, dilated bile duct is required. For *in vivo* tissue (live swine) model, biliary dilation was created by placing an endoclip, banding ligation, and argon plasma coagulation^[16]^ or using radiofrequency ablation (10 W, 80°C, 90 seconds for 2 rounds) to create biliary obstruction.^[17]^ Despite the fact that this technique could provide realistic situation, the preparation was not simple and was time-consuming. To create adequate biliary dilation, many procedures were needed to create biliary obstruction, which might be difficult due to different pig anatomy. Moreover, an *in vivo* model might be difficult for beginners.^[18]^

### Ex vivo tissue model

Because the preparation of an *in vivo* tissue model is not easily prepared, an *ex vivo* model combined with semisynthetic has been developed. Specially designed 3D-printed bile duct made from 2-mm-thick glass or polycarbonate combined with an intravenous set that simulates the blood flow was wrapped with pig or goat liver and placed in a polycarbonate box.^[18]^ The polycarbonate bile duct was drilled to create holes for EUS-guided puncture. This model provided a 4-step practice for EUS-BD, which included needle puncture, guidewire manipulation, tract dilation, and stent placement. This model could provide excellent teaching tools for novices in EUS-BD. However, a major drawback to this model is that it requires fluoroscopy. Moreover, using animal tissue requires freshly prepared material and raises the concern of zoonotic issues.

Recently, a hybrid tissue model that allows training for multiple EUS intervention procedures, such as EUS-guided fine-needle biopsy, BD, pancreatic duct drainage, pseudocyst drainage, and gastroenterostomy, has been developed.^[19]^ This model, the so-called EUS Magic Box, is the first model to allow multiple training within a single model. A video camera is used to replace the fluoroscopy. Interestingly, the main limitation to develop the model that allows the realistic sensation is the upper gastrointestinal part, which still necessitates the use of *ex vivo* porcine tissue.

### Nontissue model

**Figure 3:**
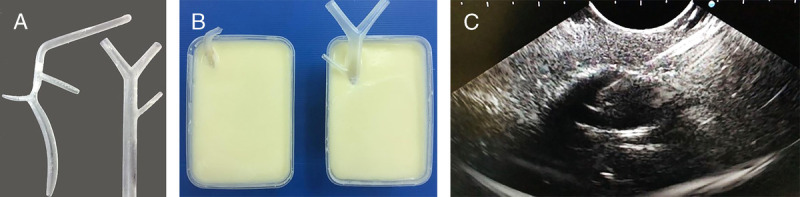
EUS-BD model using gelatin-cornstarch. The synthetic bile duct was made by 3D-printed resin (A), which was partially embedded in gelatin-cornstarch frame (B). The echo view was similar to liver parenchyma (C). BD: Biliary drainage.

**Figure 4:**
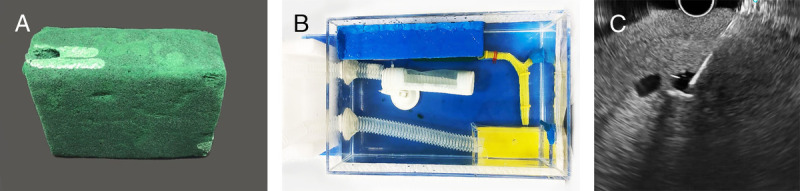
EUS-HGS model using a floral foam. The floral foam is shaped into the left lobe liver with tunnels created to represent the bile duct (A). The model is placed in acrylic box filled with water and connected to a glass tube that represents the hilar bile duct (B). When the tip of the echoendoscope is placed in the corrugate tube, segments 2 and 3 of the intrahepatic bile ducts can be seen (C). HGS: Hepaticogastrostomy.

**Figure 5:**
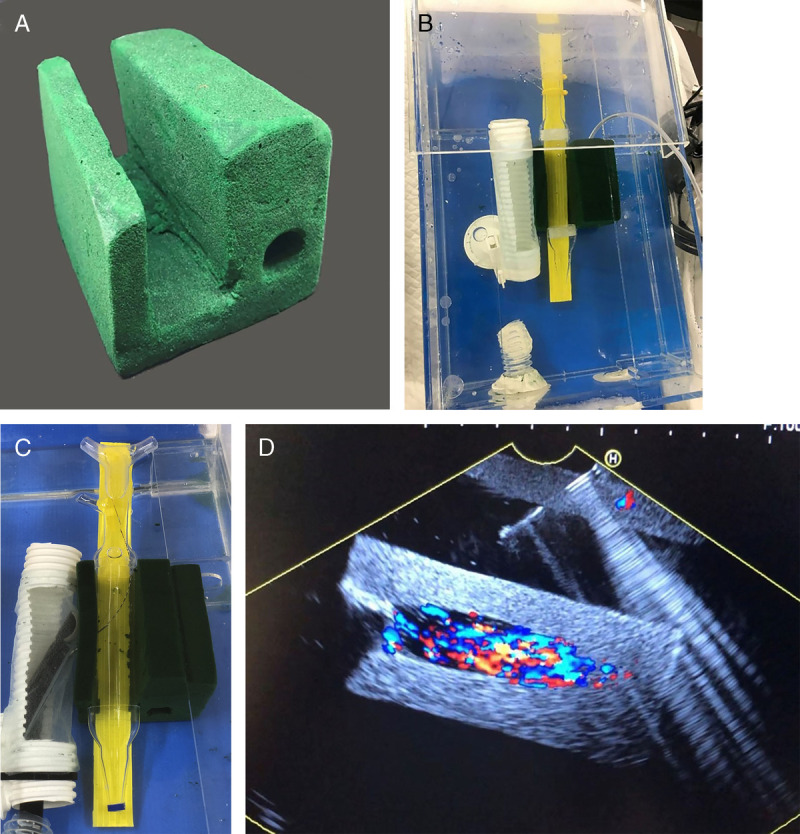
EUS-CDS model using a floral foam. The floral foam is shaped into portoduodenal soft tissue (A). A transparent plastic tube to represent the common bile duct is placed in the center along with the tunnel that represents the portal vein (B). During puncture, the instrument could be seen through the plastic tube (C), and Doppler flow could be created by pumping water through the tunnel (D). CDS: Choledochoduodenostomy.

A nontissue model for EUS-BD has been developed along with the EUS pseudocyst drainage using the same material [Figure 3].^[20]^ The synthetic bile duct was made from 3D-printed resin with holes for instrument insertion [Figure 3A] and partly embedded in cornstarch-gelatin mixture to simulate liver parenchyma [Figure 3B]. The upper end of the synthetic bile duct was visible, so there was no need for fluoroscopy. This model is fully synthetic, and the model material was cheap. However, the durability of the starch model is poor and needs to be refrigerated. Later, another model using a floral foam shaped to simulate the liver and hepatoduodenal region was developed.^[21]^ This model is durable and cheap and provides a multistep practice for both EUS-HGS [Figure 4] and EUS-CDS [Figure 5], especially for beginners. In the HGS model, the floral foam is shaped into a cubic and drilled to represent the left lobe liver [Figure 4A]. The model is embedded in an acrylic box filled with water, and the stomach is made from the corrugated tube half covered by spunbond [Figure 4B]. The hilar bile duct made from glass tube was connected to the “bile duct” opening so that the guidewire could be seen directly. When the tip of the echoendoscope is placed in the corrugated tube, segments 2 and 3 of the left intrahepatic bile duct can be seen [Figure 4C]. In the CDS model, a floral foam is shaped to represent the portoduodenal area, and another tunnel was made adjacent to the bile duct to represent the portal vein [Figure 5A]. A transparent plastic tube representing the bile duct is placed parallel to the synthetic duodenal bulb made from the corrugated tube half covered by spunbond [Figure 5B]. After EUS puncture, the instrument could be seen directly [Figure 5C], and water could be flushed through the tunnel to represent the portal vein [Figure 5D]. By the same model that includes the ampulla of Vater, the practice for EUS-guided rendezvous is also possible.

## MODEL FOR EUS-GUIDED GASTROENTERIC ANASTOMOSIS

**Figure 6:**
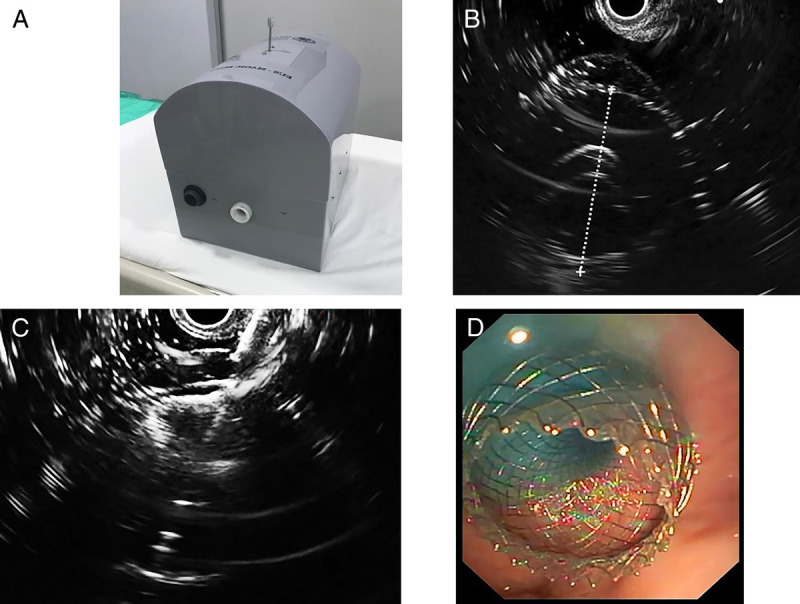
The model for EUS-guided gastroenteric anastomosis (EUS Magic Box) (A). This hybrid model provides the training for EUS-guided gastroenteric anastomosis. The echo view of the duodenum made from silicone could be seen (B) and punctured using lumen-apposing stent (C). The endoscopic view showed a fully deployed lumen-apposing stent inside the pig stomach (D). Courtesy of Professor Vinay Dhir, Raheja hospital, Mumbai, India.

EUS-guided gastroenteric anastomosis has become one of the modalities for the bypass of enteric stricture or as the portal for biliary intervention in patients who received gastric bypass surgery. The procedure feasibility and evaluation of new equipment were performed in live pigs.^[22–24]^ For the training of the procedure, so far there has been only one *ex vivo* tissue model, the EUS Magic Box, which provides the hands-on gastroenteric procedure along with other EUS-guided intervention procedures^[19]^ [Figure 6]. This model provided a pig stomach that is placed next to the duodenum made from silicone tube.

## MODEL FOR EUS-GUIDED VASCULAR INTERVENTION

EUS-guided vascular intervention has an increasing role in the diagnosis and treatment of various diseases ranging from portal venous pressure measurement to vascular embolization and shunt placement.^[25,26]^ EUS-guided portal venous measurement was initially performed in a live pig model.^[27]^ Portal hypertension was induced by injection of polyvinyl alcohol into the portal vein. Up to the present, there have been limited data regarding the development of a synthetic model for EUS-guided vascular intervention.

## THE NEED FOR FLUOROSCOPY

In a model that utilizes porcine tissue, only the endoscopic view and the echoendoscopic images can be seen, and fluoroscopy is necessary to visualize the guidewire or other instruments.^[9,19,28]^ The newly developed hybrid model provides the overhead projector, which enables the user to see the tip of the guidewire and the instruments to obviate the use for fluoroscopy.^[19]^ For a nontissue model,^[29]^ the synthetic bile duct is made of a transparent tube so that the instrument can be seen using the overhead projector instead of fluoroscopy.

### Strengths and limitations of the different types of models

*In vivo* models, *ex vivo* tissue models, or nontissue models provide different strengths and limitations. *In vivo* models provide the most realistic images and sensation as well as being the best model to demonstrate potential complications. The major drawbacks are the cost, the need for fluoroscopy, and the need for complex preparation to create abnormal anatomy prior to the procedure. Also, most of these models are not suitable for repeated procedures by multiple trainees. As a result, these models are mainly used in experiments for novel procedures. *Ex vivo* models provide less complex preparation than the *in vivo* model with less cost and allow repeated procedures. The major drawbacks are that most models require fluoroscopy, the tissue needs to be freshly prepared, and using porcine tissue raises concerns over zoonotic issues. The nontissue models are most easily prepared, need no fluoroscopy, raise no concerns over zoonotic issues, and are suitable for multiple procedures by the trainees. The major drawbacks are that these models provide the lease realistic images and sensation. As these models are less costly and suitable for repeated procedures, they are mostly used in hands-on training workshops.

## SUMMARY

EUS-guided intervention procedure has an increasing role in clinical practice. The development of a hands-on model started from a tissue model to a nontissue model, yet still has room for improvement.
